# Transfer Learning from Pre‐trained 3D Models for Enhanced Parkinson's Disease Detection

**DOI:** 10.1002/alz70856_101856

**Published:** 2025-12-25

**Authors:** Tanvi Verma, Jia Huang, Yuting Song, Mingrui Tan, Adeline Su Lyn Ng, Simon Kang Seng Ting, Shahul Hameed, Louis CS Tan, Eng King Tan, Wing Lok Au, Fei Gao, Yong Liu, Siow Mong Rick Goh, Kok Pin Ng

**Affiliations:** ^1^ Institute of High Performance Computing (IHPC), Agency for Science, Technology and Research (A*STAR), Singapore, Singapore; ^2^ National Neuroscience Institute, Singapore, Singapore

## Abstract

**Background:**

Deep learning models for Parkinson's disease (PD) diagnosis through Magnetic Resonance Imaging (MRI) face significant challenges due to limited labeled datasets. While deep learning approaches show promise, the scarcity of large‐scale annotated MRI datasets for PD detection constrains model performance. Transfer learning from models pre‐trained on large‐scale medical imaging tasks offers a potential solution by utilizing knowledge from related domains.

**Method:**

We leveraged Med3D, a 3D convolutional neural network pre‐trained on medical segmentation tasks across different modalities (MRI and CT) and diverse anatomical regions (brain, heart, prostate, spleen etc.), to extract meaningful features from volumetric medical data. The architecture was adapted for PD classification by modifying the final layers while maintaining the core network structure. To address the class imbalance in our dataset (∼20% controls, ∼80% PD), we implemented sampling techniques during training. We performed end‐to‐end fine‐tuning of the entire network using 3D brain MRI scans from the Parkinson's Progression Markers Initiative (PPMI) database. This approach allows the model to adapt its learned representations from general medical imaging tasks to the specific patterns indicative of PD in MRI data, while benefiting from the robust feature extraction capabilities developed during pre‐training.

**Result:**

We evaluated our model using the PPMI dataset, comprising 662 3D MRI scans. The dataset was split into 528 images for training, 67 for validation, and 67 for testing. Our transfer learning approach achieved 94.03% accuracy, 100% sensitivity, 60% specificity, 93.44% precision, and 0.966 F1‐score on the test set, with an AUC‐ROC of 0.8. This significantly outperformed the baseline model trained from scratch which achieved 75% accuracy. This substantial improvement demonstrates the effectiveness of utilizing pre‐trained weights for PD detection.

**Conclusion:**

Our transfer learning approach demonstrated excellent performance in PD detection, achieving 94.03% accuracy and 100% sensitivity. The significant improvement over the baseline model (75% accuracy) supports the effectiveness of utilizing pre‐trained weights from diverse medical imaging tasks. These results establish a promising framework for developing reliable computer‐aided diagnosis systems of PD and neurodegenerative diseases using MRI data.

